# Endosomal sorting and c-Cbl targeting of paxillin to autophagosomes regulate cell-matrix adhesion turnover in human breast cancer cells

**DOI:** 10.18632/oncotarget.16105

**Published:** 2017-03-10

**Authors:** Chia-Hao Chang, Krikor Bijian, Dinghong Qiu, Jie Su, Amine Saad, Michael S Dahabieh, Wilson H Miller, Moulay A Alaoui-Jamali

**Affiliations:** ^1^ Department of Medicine, Lady Davis Institute for Medical Research and Segal Cancer Center, SMBD Jewish General Hospital, Faculty of Medicine, McGill University, Montreal, Canada; ^2^ Department of Oncology, Lady Davis Institute for Medical Research and Segal Cancer Center, SMBD Jewish General Hospital, Faculty of Medicine, McGill University, Montreal, Canada

**Keywords:** paxillin, focal adhesion dynamics, Rab7-GTPase, c-Cbl, autophagy

## Abstract

Post-translational mechanisms regulating cell-matrix adhesion turnover during cell locomotion are not fully elucidated. In this study, we uncovered an essential role of Y118 site-specific tyrosine phosphorylation of paxillin, an adapter protein of focal adhesion complexes, in paxillin recruitment to autophagosomes to trigger turnover of peripheral focal adhesions in human breast cancer cells. We demonstrate that the Rab-7 GTPase is a key upstream regulator of late endosomal sorting of tyrosine118-phosphorylated paxillin, which is subsequently recruited to autophagosomes via the cargo receptor c-Cbl. Essentially, this recruitment involves a direct and selective interaction between Y118-phospho-paxillin, c-Cbl, and LC3 and is independent from c-Cbl E3 ubiquitin ligase activity. Interference with the Rab7-paxillin-autophagy regulatory network using genetic and pharmacological approaches greatly impacted focal adhesion stability, cell locomotion and progression to metastasis using a panel of human breast cancer cells. Together, these results provide novel insights into the requirement of phospho-site specific post-translational mechanism of paxillin for autophagy targeting to regulate cell-matrix adhesion turnover and cell locomotion in breast cancer cells.

## INTRODUCTION

Cell migration is a physiological process fundamental for embryonic development, immune and inflammatory responses, wound healing, and tissue homeostasis [1, 2]. Aberrant cell migration has been associated with several pathologies including rare inherent diseases such periventricular heterotopia [3], Baraitser-Winter syndrome [4] and more common diseases such as cancer progression to metastasis [5].

In general, motile cells exhibit finger-like protrusions (e.g. lamellipodia, filopodia) of the plasma membrane that are stabilized by the formation of focal adhesions (FAs). The synchronous cycle of FA assembly/disassembly are essential for cell locomotion [6] and require the integration of multiple signals from extracellular matrix-receptor interactions, cell cytoskeleton and binding to intracellular proteins primarily those involved in endocytic trafficking, in particular members of the Rab GTPase family involved in early endosome development [7–11], many of which are amplified in invasive cancers and have been associated with enhanced cancer cell invasiveness [12–14]. However, there remains a fundamental gap in understanding the molecular mechanisms by which Rab GTPases that are involved in late endosomal maturation regulate FA turnover, with a particular focus on FA posttranslational modifications that trigger these events.

In this study, we report a novel role for the late endosomal protein Rab7 GTPase in the regulation of the selective recruitment of tyrosine 118 (Y118)-phosphorylated paxillin, but not Y31-phospho-paxillin to autophagosomes via its interaction with c-Cbl, which serves as a cargo receptor independently of its E3 ubiquitin ligase activity, in human breast cancer cells. This novel Rab7-mediated turnover of 118Y-p-paxillin was inhibited upon Atg12 downregulation, thereby reinforcing the implication of the autophagic pathway. This regulatory axis was not seen with other FA proteins such as FAK and was not affected by Src manipulation. Although autophagy-mediated FA turnover has recently been identified in mouse mammary 4T1 cells [15], studies describing this mechanism in human cells have yet to be elucidated. Therefore, these novel interactions which are required for the motility and invasiveness of human breast cancer cells, may serve as an effective approach for targeting the progression of this disease.

## RESULTS

### Rab7 regulates FA turnover, cancer cell locomotion and progression to metastasis

**Figure 1:**
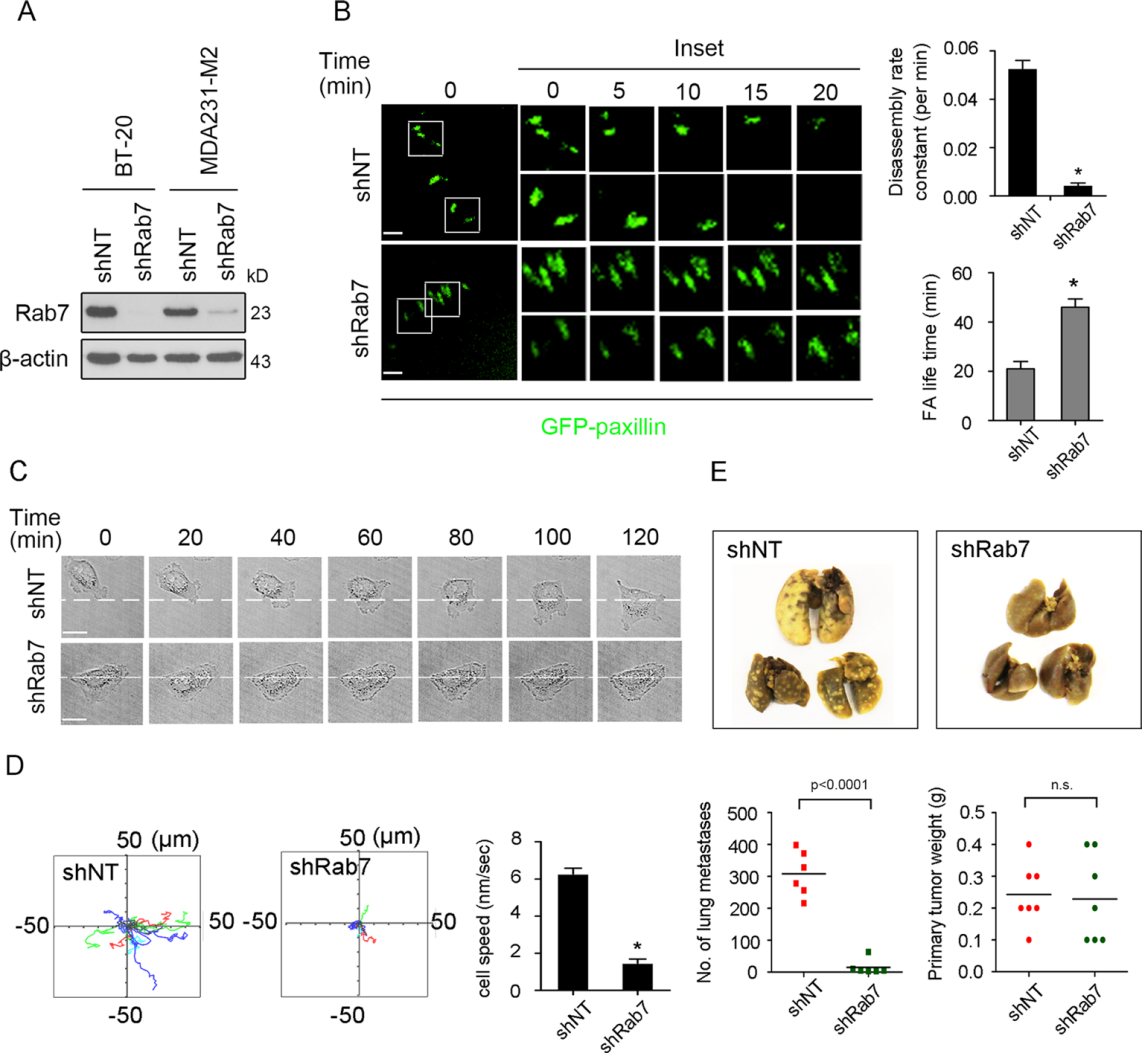
Knockdown of Rab7 decreases focal adhesion turnover, cell migration and progression to metastasis (**A**) MDA231-M2 and BT-20 cells were lysed and immunoblotted with Rab7 and β-actin antibodies. β-actin was used as an internal control. (**B**) Left: Serum- starved BT-20 cells expressing non-targeted shRNA (shNT) and Rab7 shRNA (shRab7) were transfected with GFP-paxillin for 24 h and focal adhesion (FA) turnover was analyzed by time-lapse spinning disc microscopy with 2 μm scale bar upon 20 ng/ml EGF stimulation. Higher-magnification images of the inserts are shown indicating positions of paxillin-containing FAs. Right: Quantification of FA disassembly and FA duration of shNT- and shRab7-expressing cells. Data are presented as mean ± SEM (*p < 0.05, *n* = 53 FAs in shNT and *n* = 62 FAs in shRab7 groups from 10 single cells). (**C**) Serum-starved shNT- and shRab7-MDA-231-M2 cells were stimulated with 20 ng/ml EGF and then cell motility monitored by time-lapse spinning disc microscopy. Scale bar, 20 μm. (**D**) Left: The paths of single MDA-231-M2 of shNT and shRab7 were tracked for 2 hours at a rate of 1 frame per 7.5 minutes. 15 tracks of shNT- and shRab7-expressing cells were plotted in different colors, respectively. Right: Speed quantification of MDA-231-M2 cells expressing shNT or shRab7 (mean ± SEM, *n* = 15 cells, *p < 0.05) (**E**) Top, Graphs show the lung with metastatic nodules from the mice implanted with shNT or shRab7 MDA231-M2 cells. Bottom, quantification of the mean number of lung metastasis and the weight of primary tumor in mammary fat pad (mean ± SEM, *n* = 6 SCID mice, *p < 0.05).

To understand the role of the Rab7 GTPase in cancer cell migration, we first investigated its impact on FA turnover using two human breast cancer cell lines with intrinsic invasive properties, MDA231-M2 and BT-20 cells, and their matched counterparts where Rab7 was stably knockdown by shRNA (Figure 1A). Time-lapse confocal imaging in BT-20 cells expressing GFP-tagged paxillin revealed slower FA disassembly and persistence of FAs in Rab7-silenced cells compared to control cells (Figure 1B, left and quantification in the right panel). A similar observation was made using the nocodazole-based assay to enable transient synchronization of FA disassembly (revealed by an enrichment of FA formation as the result of exposure to nocodazole for 2 hours followed by FA disassembly and recovery after nocodazole washout [16]. Under this condition, maximal FA disassembly was seen at 30 min post-nocodazole washout, followed by reassembly of FAs, which was obvious from 30 min to 1 h. In comparison to control cells, Rab7-silenced cells reveal slower turnover of 118Y-p-paxillin, especially at 15 min after nocodazole washout (Supplementary Figure 1).

Investigation of cell migration confirmed that in MDA231-M2 cells where Rab7 was downregulated, cell locomotion was significantly compromised compared to control cells (Figure 1C, 1D and Supplementary Video 1). Similar results were seen in BT-20 cells. Noteworthy, reduced cell locomotion was not mediated by changes in cell proliferation as no difference in cell growth was seen between Rab7-shRNA and their matched control cells (Supplementary Figure 2).

To further confirm the correlation between these *in vitro* observations and cancer progression *in vivo*, we investigated the impact of Rab7 down-regulation on cancer cell progression to metastasis using MDA231-M2 cells expressing control shNT or Rab7 shRNA implanted orthotopically into the mammary fat pad of SCID mice. After 40 days observation, a significant inhibition in the number of lung metastases was seen with the Rab7-silenced cells compared to control cells (Figure 1E, top). Quantification confirmed that the number of lung metastases were significantly decreased in the absence of Rab7 (Figure 1E, bottom), despite having similar primary tumors weights.

### Knockdown of Rab7 promotes paxillin enrichment in cytoplasmic puncta

**Figure 2:**
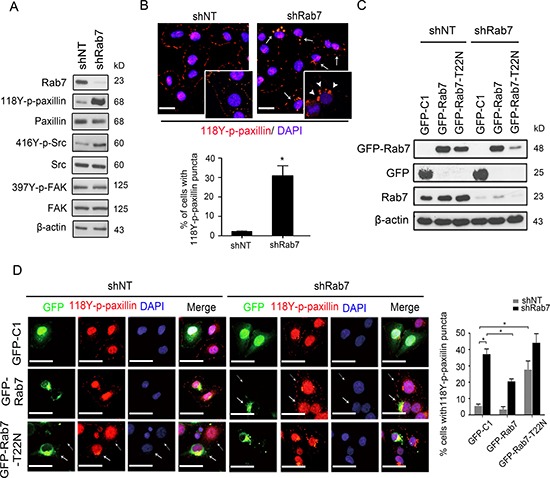
Rab7 and its GTPase activity are required for paxillin accumulation into cytosolic puncta (**A**) Lysates from shNT or shRab7-expressing BT-20 cells were immunoblotted with antibodies against Rab7, LC3B, 118Y-p-paxillin, paxillin, 397Y-p-FAK, FAK416Y-p-Src, Src and β-actin as an internal control. (**B**) Top: shNT and shRab7 transfected BT-20 cells were fixed and stained with anti-118Y-p-paxillin antibody (red) and with DAPI (blue). Solid arrows identify cells with 118Y-p-paxillin puncta. Arrowheads in inserts (65x magnification) indicate actual 118Y-p-paxillin puncta. Bottom: percentage of cells with 118Y-p-paxillin puncta. Data are presented as mean ± SEM (*p < 0.05, *n* = 3). (**C** and **D**) BT-20 cells expressing shNT and shRab7 plasmids and their matched cells rescued with empty (GFP-C1), shRNA-resistant Rab7 (GFP-Rab7) or Rab7 with a point mutation (GFP-Rab7-T22N) plasmids, were lysed and immunoblotted with anti-GFP antibody (C) or were fixed and stained with anti-118Y-p-paxillin antibody (red) and with DAPI (blue). Scale bar, 20 μm. Solid arrows indicate the cell expressing GFP plasmids and dashed arrows indicate cells without expressing GFP plasmids (D, left). (D, right) Graph shows the quantification of percentage of cells with 118Y-p-paxillin in intracellular puncta (determined using lower magnification images (20 ×)). Data are presented as mean ± SEM (*P < 0.05, *n* = 3)

To further investigate the relationship between Rab7 expression and FA dynamics we examined the protein levels of various FA components in BT-20 cells under conditions where Rab7 is downregulated. As such, we observed increasing levels of phosphorylated paxillin and Src in Rab7 shRNA cells, compared to control cells (Figure 2A). Other FA-associated proteins such as FAK remained unchanged. Furthermore, we observed pronounced relocation of 118Y-p-paxillin to distinctive intracellular puncta in cells where Rab7 was downregulated, while 118Y-p-paxillin predominantly localized to adhesion sites in control cells (Figure 2B, top and quantified in the bottom panel).

To investigate if Rab7-GTPase activity was essential for paxillin relocalization into these cytoplasmic puncta, we expressed control (GFP-C1), wild-type Rab7 (GFP-Rab7) or a Rab7-GTPase defective mutant (GFP-Rab7-T22N) [17] in control and Rab7-silenced cells (Figure 2C). As shown in Figure 2D (solid arrows), in Rab7-deficient cells where the expression of wild type Rab7 was restored, the expression of 118Y-p-paxillin in FAs was rescued. However, expression of the dominant negative GFP-Rab7-T22N resulted in the reappearance of perinuclear 118Y-p-paxillin puncta even in control cells expressing endogenous Rab7 (Figure 2D, left and quantification in the right panel). These findings demonstrate that interfering with Rab7 or its GTPase activity prevented the trafficking of phosphorylated paxillin.

### 118Y-p-paxillin accumulates in autophagolysosomes in Rab7-depleted cells

**Figure 3:**
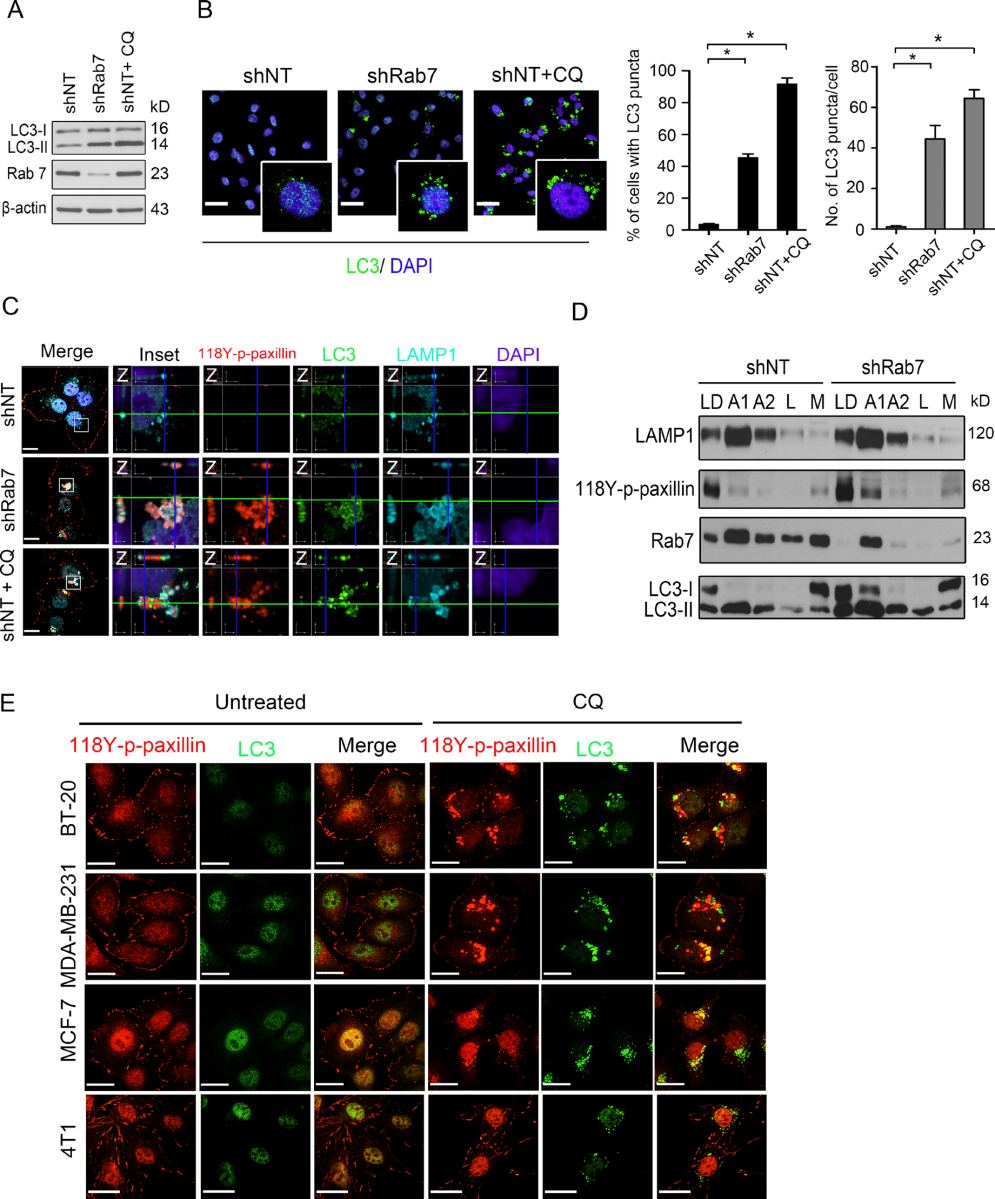
Knockdown Rab7 mimics the effect of chloroquine to localize paxillin into autophagolysosomes (**A**–**C**) Control (shNT), Rab7-silenced (shRab7) and control treated with 20 μM chloroquine (CQ) for 24 h (shNT+CQ) BT-20 cells lysed and immunoblotted with antibodies against Rab7, LC3B and β-actin (internal control) (A), fixed and stained with anti-LC3 antibody (green) and with DAPI (blue) with 20 μm scale bar left (insert 63X) and quantification on the right panel showed percentage of cells with LC3 puncta and average # of LC3 puncta/cell (*N* = 15) (B). Data are presented as mean ± SEM (*p < 0.05, *n* = 6)) or further co-stained with anti-118Y-p-paxillin antibody (red), anti-LC3B antibody (green), anti-LAMP-1 antibody (cyan) and with DAPI (blue) with 20 μm scale bar (C). Enlargements and cross-sections of the confocal-z-planes of the boxed regions are also shown indicating association of 118Y-p-paxillin, LC3B and LAMP-1. (**D**) Lysed shNT and shRab7-expressing BT-20 cells were subjected to nycodenze gradient centrifugation, then analyzed by immunoblotting using anti-LAMP1, anti-118Y-p-paxillin, anti-Rab7 and anti-LC3 antibodies. LD is the loading control, A1 and A2 are autophagosome-related fractions, L is the lysosomal fraction and M is the mitochondrial fraction. (**E**) MDA-MB-231, BT-20 and MCF-7 cells were treated either with PBS (untreated) or CQ for 24 h, then fixed and stained with anti-118Y-p-paxillin or anti-416Y-p-Src antibodies (red) and anti-LC3 antibody (green). Scale bar, 20 μm.

Rab7 plays an essential role in the maturation of late autophagic vacuoles [18, 19]. Therefore, we investigated whether 118Y-p-paxillin was arrested in these late autophagic vacuoles. To do so, we first used chloroquine (CQ), a small molecule that accumulates in autophagic vesicles to prevent fusion of autophagosomes to lysosomes [20]. As shown in Figure 3A, exposure of cells to CQ for 24 h significantly led to the accumulation of LC3-II, which was similar to what we observed in cells expressing Rab7 shRNA (Figure 3A). Moreover, both CQ and Rab7 shRNA induced LC3 puncta formation, as compared to respective controls (Figure 3B), which indicated that both approaches cause late stage autophagy blockade. To further decipher the localization of these 118Y-p-paxillin puncta, co-staining of 118Y-p-paxillin with LAMP-1 (lysosome marker) and LC3 (autophagy marker) was performed. As shown in Figure 3C, the puncta observed upon Rab7 knockdown or CQ treatment were indicative of an accumulation in autophagolysosomes (Figure 3C). These findings were further supported by our density gradient centrifugation studies, which consisted of enriching various cellular compartments including autophagosomes. Although 118Y-p-paxillin accumulation in autophagosomes is clearly visible in both cell lines, increased accumulation is observed in shRab7 autophagosomes since trafficking is compromised in this condition (Figure 3D). Furthermore, monitoring FA dynamics in live cells revealed significantly reduced FA disassembly rates and prolonged FA duration at FA site in CQ-treated cells, as compared to controls (Supplementary Figure 3). This specific localization of 118Y-p-paxillin with LC3 in autophagosomal puncta upon CQ treatment was not exclusive to BT-20 cells, as it was also observed in CQ-treated MDA-MB-231 and MCF-7 cells (Figure 3E).

### Rab7 regulates 118Y-p-paxillin turnover through the autophagy pathway rather than endosomal degradation

**Figure 4:**
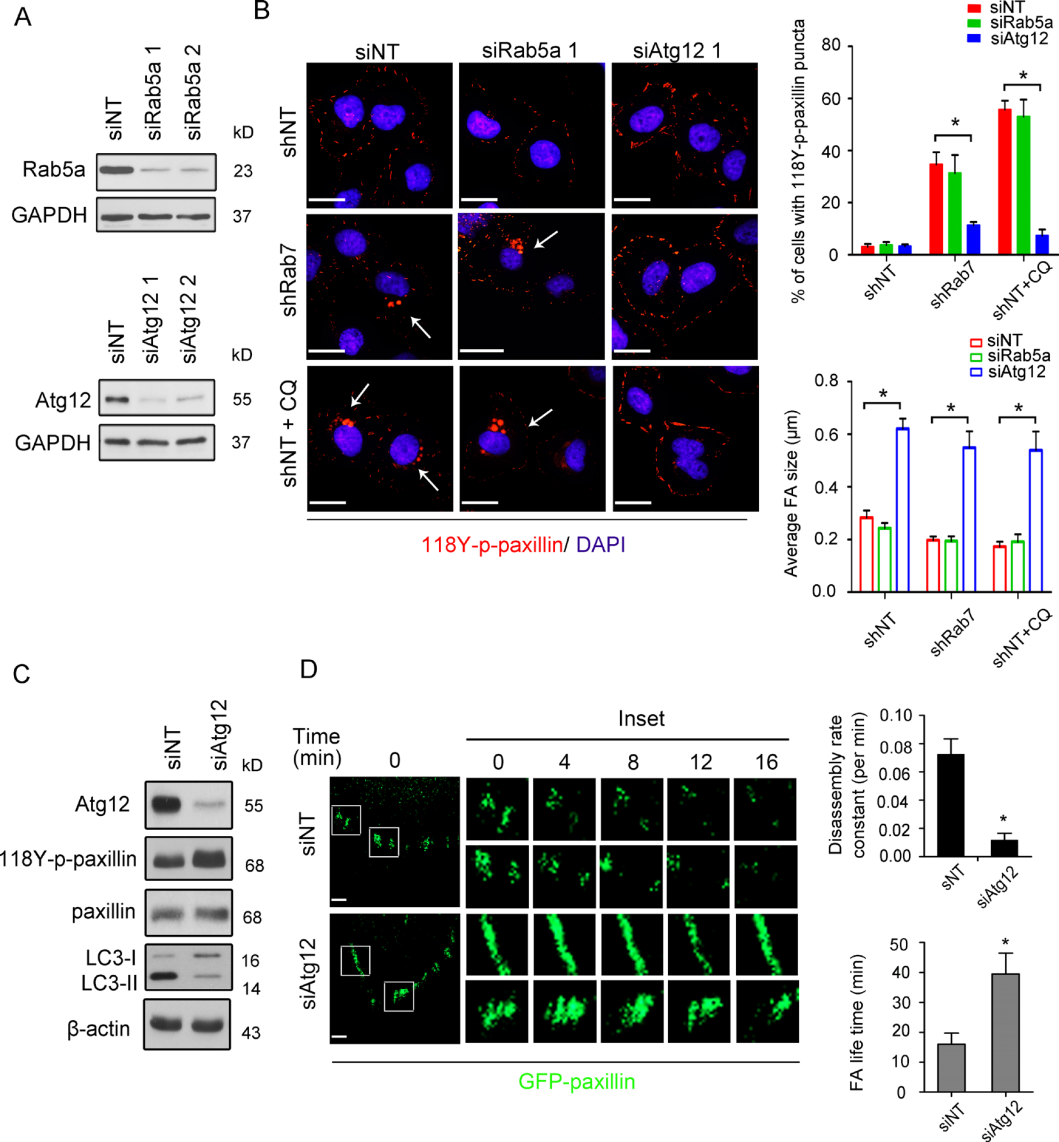
The autophagic pathway regulates Rab7-mediated paxillin turnover (**A**) BT-20 cells were transiently transfected with 100 nM non-targeted (siNT), Rab5a (siRab5) or Atg12 (siAtg12) siRNA for 48 h and total protein was extracted then immunoblotted with antibodies against Rab5a, Atg12 and anti-GAPDH as an internal control. (**B**) Left, shNT, shRab7 and shNT treated with CQ (shNT+CQ) BT-20 cells were respectively transfected with non-targeted, Rab5a or Atg12 siRNA for 48 h and stained with anti-118Y-p-paxillin antibody (red) and DAPI (blue). Arrows indicate the cells with 118Y-p-paxillin puncta. Scale bar, 20 μm. Right top, quantification of the percentage of cells with 118Y-p-paxillin puncta. Data are presented as mean ± SEM (*p < 0.05, *n* = 3). Right bottom, quantification of average of FA size. Data are presented as mean ± SEM (*p < 0.05, *n* = 10 cells) (**C**) shNT BT-20 cells were transfected with either 100nM siNT or siAtg12 for 48 h, then immunoblotted with antibodies against Atg12, 118Y-p-paxiilin, paxillin, Rab7, LC3B and anti-β-actin used as an internal control. (**D**) Left: Serum-starved siNT or siAtg12 BT-20 cells transfected with GFP-paxillin for 24 h were plated on chamber slide, stimulated by 20 ng/ml EGF and then analyzed for FA turnover by time-lapse spinning disc microscopy. Scale bar, 2 μm. Higher-magnification images of the inserts are also shown indicating positions of paxillin-containing FAs. Right: FAs disassembly and FA life time in siNT and siAtg12 cells were quantified (mean ± SEM, *n* = 75 FAs in siNT and 88 FAs in siAtg12 from 10 single cells, *p < 0.05).

Considering that Rab7 can play a role in both endosomal and autophagic pathways which converge at late autophagic vacuoles, we seeked to identify which of these pathways is primarily responsible for 118Y-p-paxillin trafficking to autophagosomes. Therefore, we silenced either Rab5 (endosomal upstream regulator) or Atg12 (autophagic upstream regulator) using siRNA (Figure 4A) and then monitored 118Y-p-paxillin puncta staining. As shown in Figure 4B, the 118Y-p-paxillin puncta staining could be rescued in Rab7-silenced cells after knocking down Atg12, but not Rab5. In addition, knockdown of Atg12 resulted in the enhancement of focal adhesion structures both in control and Rab7-silenced cells. This 118Y-p-paxillin enhancement in Atg12-silenced cells was further confirmed by immunoblotting (Figure 4C). To further confirm the dependence of FA turnover on the autophagic pathway, we monitored FA dynamics in Atg12-knockdown cells expressing GFP-paxillin. As expected, Atg12 downregulation significantly decreased the rate of paxillin disassembly (Figure 4D), further reinforcing the implication of the autophagic pathway in Rab7-mediated turnover of 118Y-p-paxillin.

### Phosphorylation of the Y118-residue of paxillin is exclusively required for its targeting to autophagosomes and for autophagy-mediated FA turnover

**Figure 5:**
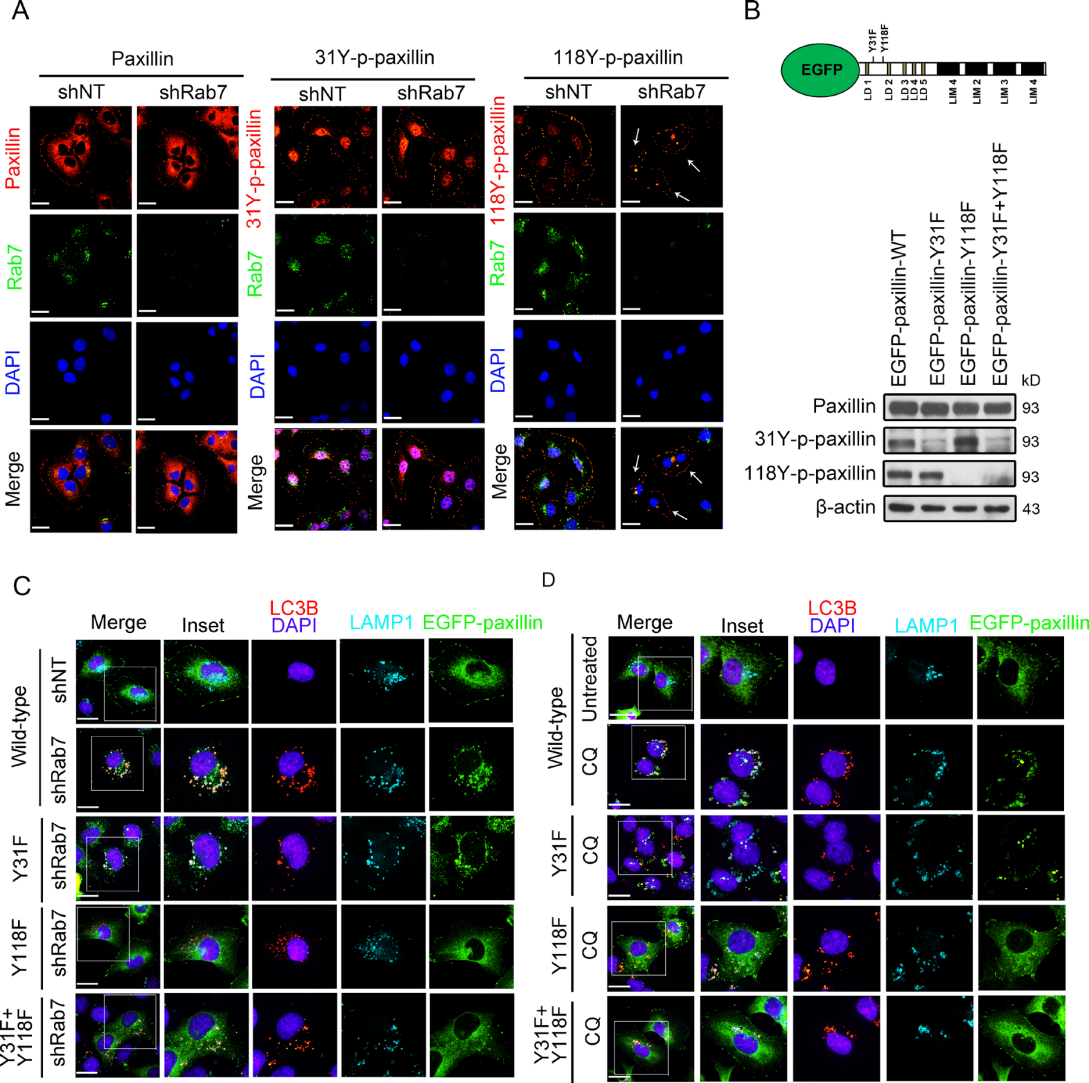
Tyrosine 118 phosphorylation of paxillin is essential for Rab7-mediated FA turnover via autophagy (**A**) BT-20 cells expressing shNT or shRab7 were fixed and stained for anti-paxillin, anti-31Y-p-paxillin or 118Y-p-paxillin (red), anti-Rab7 antibody (green) and with DAPI (blue). Arrows indicate the cells with puncta. Scale bar, 20 μm. (**B**) Top: schematic illustration of the structure of paxillin and position of tyrosine 31 and 118. Bottom: lysates from BT-20 cells transfected with EGFP-paxillin WT, EGFP-paxillin-Y31F, EGFP-paxillin-Y118F or EGFP-paxillin-Y31F/Y118F were immunoblotted with antibodies against paxillin, 31Y-p-paxillin, 118Y-p-paxillin and anti-β-actin as an internal control. (**C** and **D**) Control and (shNT) Rab7-silenced BT-20 cells (shRab7) (C) or BT-20 control (untreated) and treated with chloroquine for 24 hr (CQ) (D) were transfected with EGFP-paxillin WT, EGFP-paxillin-Y31F, EGFP-paxillin-Y118F or EGFP-paxillin-Y31F+Y118F plasmids (all green) respectively. Then, cells were immunostained with anti-LC3B antibody (red), anti-LAMP-1 antibody (cyan) and with DAPI (blue). Scale bar, 20 μm. Enlargements of the boxed regions are also shown indicating association of EGFP-paxillin, LC3B and LAMP-1.

To establish the importance of paxillin post-translational tyrosine modifications necessary for Rab7- mediated autophagosomal targeting, we investigated the two tyrosine residues previously established to be phosphorylated in dynamic focal adhesions [21], namely Tyr 31 and Tyr118. First we investigated whether non-phosphorylated paxillin or 31Y-p-paxillin can also accumulate in intracellular puncta similar to 118Y-p-paxillin in Rab7 knockdown cells. Immunocytochemistry results supported that knockdown of Rab7 only caused accumulation of 118Y-p-paxillin into puncta, whereas non-phosphorylated paxillin and 31Y-p-paxillin did not (Figure 5A). Next, we expressed EGFP-plasmids containing WT, Y31F, Y118F and Y31F/Y118F-paxillin constructs in BT20 cells (Figure 5B) and then monitored GFP-expression along with LAMP-1 and LC3B in cells where autophagy was inhibited either using Rab7 knockdown or CQ treatment. While cells expressing WT or Y31F-paxillin demonstrated similar puncta, especially at locations where LC3B and LAMP-1 colocalize, Y118F or Y31F/Y118F constructs were absent from these puncta (Figure 5C, 5D), further supporting the importance of Y118 phosphorylation for autophagosome targeting and subsequent accumulation in autophagolysosomes.

### Y118-p-paxillin interacts with LC3 at FA sites

**Figure 6:**
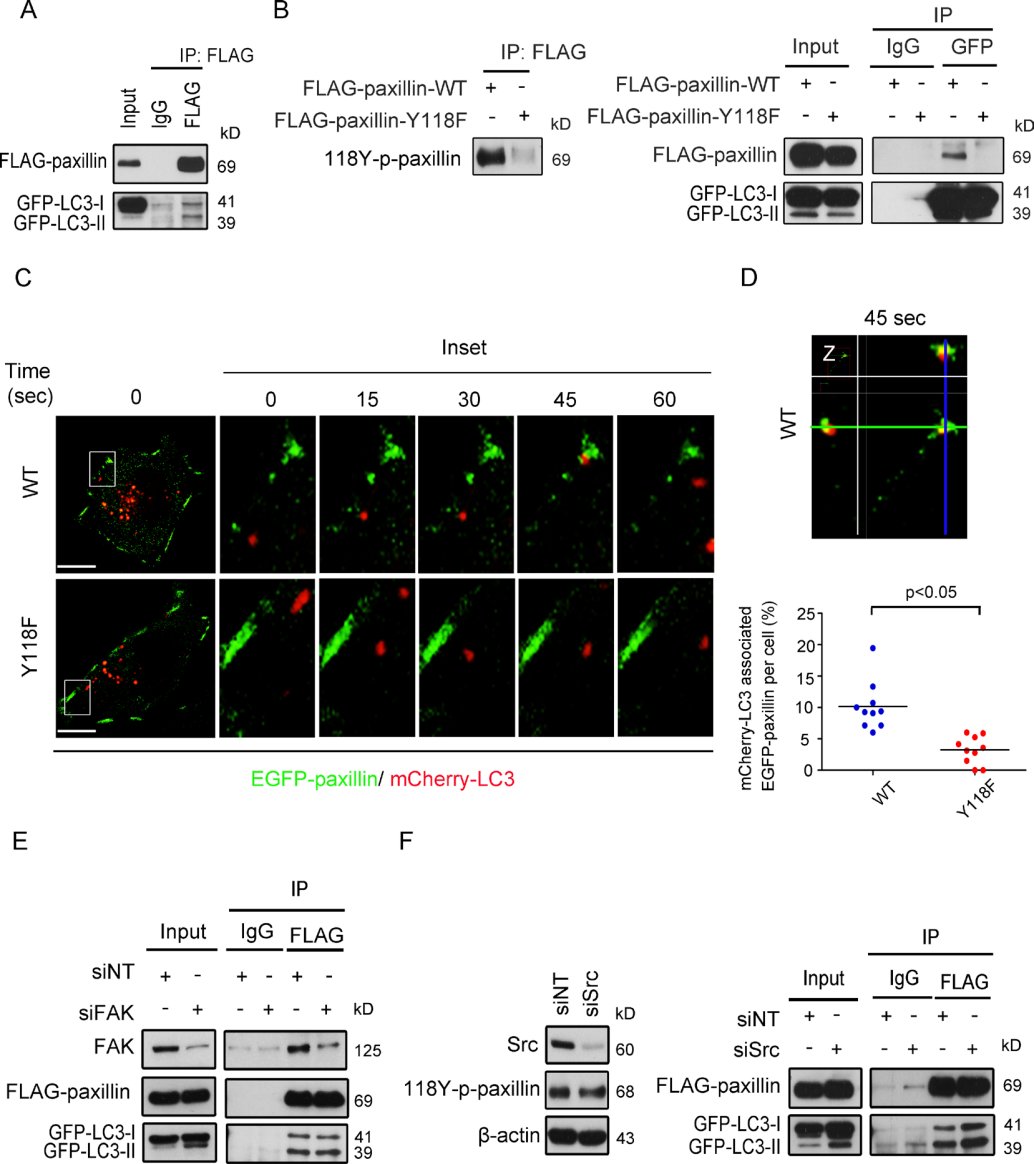
Tyrosine 118 phosphorylation of paxillin modulates its association with the autophagic marker LC3 at cell plasma membrane protrusions (**A**) BT-20 cells were transfected with FLAG-paxillin and GFP-LC3 plasmids for 24 h and immunoprecipitated with either anti-IgG or anti-FLAG antibodies. Lysates were then immunoblotted with anti-FLAG and anti-GFP antibodies. (**B**) Left: FLAG protein was immunoprecipitated from BT-20 cells transfected with either wild-type FLAG-paxillin (FLAG-paxillin-WT) or FLAG-paxillin with point mutation at amino acid 118 (FLAG-paxillin-Y118F) for 24 hours and immunoblotted with anti-118Y-p-paxillin antibody. Right: BT-20 cells were transfected with either FLAG-paxillin-WT or FLAG-paxillin-Y118F and GFP-LC3 plasmids for 24 h and immunoprecipitated with either anti-IgG or anti-GFP antibodies. Lysates were then immunoblotted with anti-FLAG and anti-GFP antibodies. (**C**) BT-20 cells were transfected with either EGFP-paxillin wild-type (WT) or EGFP-paxillin-Y118F (Y118F) plasmids (green) and mCherry-LC3 plasmids (red) for 24 h, then plated on chamber slide. Next, cells were monitored for the trafficking of EGFP-paxillin and mCherry-LC3 by time-lapse spinning disc microscopy. Scale bar, 5 μm. Higher-magnification images of the inserts are also shown indicating path of EGFP-paxillin and mCherry-LC3 at cell protrusions. (**D**) Top, Enlargements and cross-sections of the confocal-z-planes of the boxed regions are also shown indicating association of EGFP-paxillin-WT and mCherry-LC3 at 45 sec. Bottom: quantification of the percentage of dynamic EGFP-paxillin labeling FAs per cell targeted by mCherry-LC3. Scatter plot shows individual single cells and mean line. *n* = 125 mCherry-LC3 vesicles targeted to FA in EGFP-paxillin WT and 169 mCherry-LC3 vesicles targeted to FAs in EGFP-paxillin-Y118F group from 10 single cells. (**E**) siRNA silenced FAK BT-20 cells were transfected with FLAG-paxillin and GFP-LC3 plasmids for 24 h and immunoprecipitated by either anti-IgG or anti-FLAG antibodies. Lysates were then immunoblotted with anti-FAK, anti-FLAG and anti-GFP antibodies. (**F**) Left: BT-20 cells were transiently transfected with non-targeted or Src siRNA proteins were extracted, and then immunoblotted with anti-Src, anti-118Y-p-paxillin and anti-β-actin antibodies. Right: FLAG-paxillin was immunoprecipitated from siNT and siSrc cells expressing FLAG-paxillin and GFP-LC3, then immunoblotted with anti-FLAG and anti-GFP antibodies.

In order to gain further insight into mechanisms of paxillin recruitment to autophagosomes, we co-transfected BT-20 cells with FLAG-paxillin and GFP-LC3, followed by immunoprecipitation of FLAG-paxillin. As shown in Figure 6A, we were able to co-immunoprecipitate GFP-LC3 with FLAG-paxillin. Once again, this interaction was dependent on the phosphorylation of paxillin at Y118 since expression of the paxillin mutant Y118F, which is unable to get phosphorylated (Figure 6B, left), failed to interact with LC3 (Figure 6B, right). Furthermore, we co-transfected BT-20 cells with GFP-paxillin and mCherry-LC3 which allowed us to follow their intracellular localization in live cell conditions. As shown in Figure 6C, 6D) and Supplementary Video 2, transient co-localization of EGFP-paxillin-WT and mCherry-LC3 are seen at FA sites. To further confirm that this transient interaction occurs during FA disassembly, we utilized the nocodazole-based assay as previously described [22]. As expected, immunocytochemistry results demonstrated transient colocalization between 118Y-p-paxillin and LC3 at plasma membrane protrusions 10 min post nocodazole washout (Supplementary Figure 4). Likewise, expression of Y118F-paxillin resulted in significantly lower incidence of this co-localization with LC3, along with its inability to specifically co-localize at FA site (Figure 6C, bottom). Equally important, siRNA knockdown of FAK and Src prior to co-immunoprecipitation studies had no impact on paxillin/LC3 interactions (Figure 6E and 6F), supporting that the autophagy-mediated regulation of paxillin turnover is independent of FAK or Src kinases, both of which are implicated in the phosphorylation of paxillin [21].

### c-Cbl is the main cargo receptor targeting Y118-p-paxillin to autophagosomes

**Figure 7:**
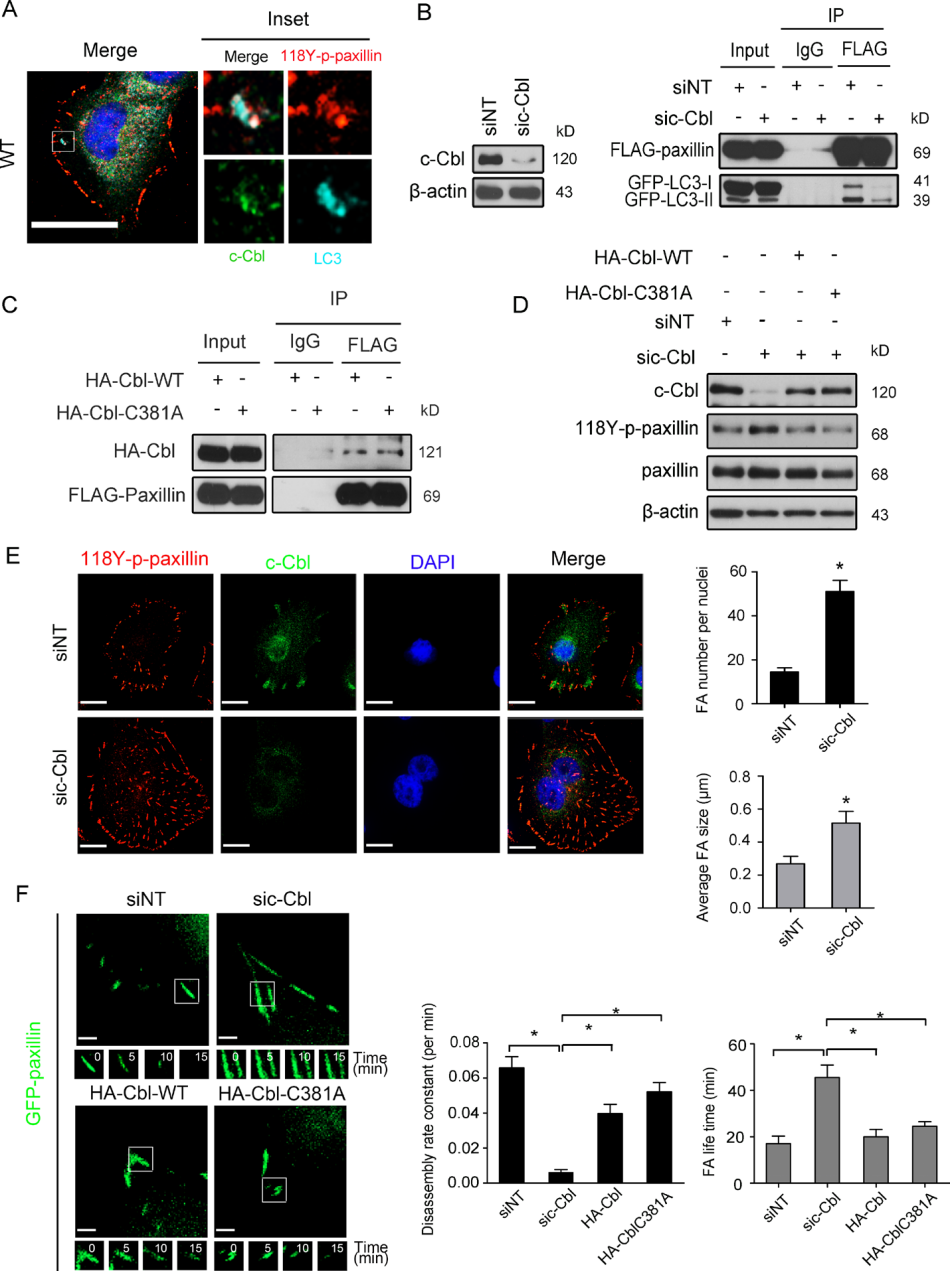
c-Cbl is the major cargo receptor mediating LC3 interaction with paxillin (**A**) BT-20 cells were fixed and stained for anti-118Y-p-paxillin antibody (red), anti-LC3B antibody (cyan), anti-c-Cbl antibody (green) and with DAPI (blue). Scale bar, 20 μm. Enlargements of the boxed regions are also shown indicating association of 118Y-p-paxillin, LC3B and c-Cbl. (**B**) Left: BT-20 cells were transfected with either NT or c-Cbl siRNA for 48 h, then immunoblotted with anti-c-Cbl anti-β-actin antibodies. Right: BT-20 cells were transfected with non-targeted or c-Cbl siRNA for 24 h and then transfected with FLAG-paxillin and GFP-LC3 plasmids for another 24 h and immunoprecipitated by either anti-IgG or anti-FLAG antibodies. Lysates were then immunoblotted with anti-FLAG and anti-GFP antibodies. (**C**) BT-20 cells were transfected with either HA-Cbl wild-type plasmids (HA-Cbl-WT) or HA-Cbl with point mutation on a.a 381. (HA-Cbl-C381A) for 24 h and immunoprecipitated by either anti-IgG or anti-FLAG antibodies. Lysates were then immunoblotted with anti-FLAG and anti-HA antibodies. (**D**) BT-20 cells were transfected with non-targeted or c-Cbl siRNA for 24 h and then were either rescued by HA-Cbl-WT or HA-Cbl-C381A plasmids. After 24 h, cells were lysed and immunoblotted with antibodies against c-Cbl, 118Y-p-paxillin, paxillin and anti- β-actin as an internal control. (**E**) Left, BT-20 cells transfected with non-targeted or c-Cbl siRNA for 48 h were stained with anti-118Y-p-paxillin antibody (red), c-Cbl antibody (green) and DAPI (blue). Scale bar, 20 μm. Right, quantification of FA number per cells and average FA size. Data are presented as mean ± SEM (*p < 0.05, *n* = 10 cells) (**F**) Left: Serum-starved siNT or sic-Cbl BT-20 cells rescued with either HA-Cbl wild-type plasmids (HA-Cbl-WT) or HA-Cbl with point mutation on a.a 381 (HA-Cbl-C381A) were transfected with GFP-paxillin for 24 h, then plated on chamber slide, stimulated by 20 ng/ml EGF and analyzed for FA turnover by time-lapse spinning disc microscopy. Scale bar, 2 μm. Higher-magnification images of the inserts are also shown indicating positions of paxillin-containing FAs. Right: FAs disassembly rate and FA life time in siNT, sic-Cbl. sic-Cbl+ HA-Cbl-WT and sic-Cbl+ HA-Cbl-C381A cells were quantified (mean ± SEM, *n* = 44 FAs in siNT, 38 FAs in sic-Cbl, 45 FAs in HA-Cbl rescued and 52 FAs in HA-Cbl-C381A groups from 10 single cells, *p < 0.05).

The E3 ubiquitin ligase activity of c-Cbl, via its LIR (light chain 3 (LC3)-interacting region) domain, has been reported to recruit Src to LC3 [23]. Additionally, c-Cbl has been reported to specifically interact with paxillin [24]. Therefore, in order to explore whether c-Cbl mediates the LC3/paxillin interaction, we used immunofluorescence staining to investigate the association. We noticed the presence and co-localization of a c-Cbl/118Y-p-paxillin/LC3 complex at focal adhesions (Figure 7A). Next, we knocked down c-Cbl expression using siRNA (Figure 7B, left) and then pulled down the complex. As shown in Figure 7B (right panel), c-Cbl siRNA expression considerably reduced LC3 binding to paxillin. This interaction was independent of the E3 ubiquitin ligase activity of c-Cbl, as expression of a E3 ubiquitin ligase- defective mutant of c-Cbl (HA-Cbl-C381A) still maintained an effective interaction with paxillin (Figure 7C). Interestingly, c-Cbl siRNA also caused the accumulation of 118Y-p-paxillin in BT-20 cells, an accumulation that can be reversed by the re-expression of either WT or C381A c-Cbl (Figure 7D). The significance of this accumulation was visualized by immunofluorescence, where enhanced 118Y-p-paxillin structures (density and number) were observed in BT-20 cells where c-Cbl was knocked down, as compared to control cells (Figure 7E). Furthermore, monitoring of EGFP-paxillin turnover in motile cells revealed lower disassembly rates in BT-20 cells where c-Cbl is silenced, which was once again effectively rescued by re-expression of WT or C381A c-Cbl (Figure 7F).

## DISCUSSION

Cell locomotion is controlled by actin cytoskeleton-associated adhesions, represented by a network of proteins among which the scaffolding protein paxillin plays a fundamental regulatory role in the formation of both nascent and mature focal adhesions at leading edges of motile cells [25–27]. These functions are attributed to multiple interactions of paxillin with FA partners, primarily involving its zinc-finger motifs and are tightly regulated by paxillin posttranslational modifications, including phosphorylations at tyrosine residues 31 and 118 [28–31]. The role of these phosphorylation sites in the regulation of FA dynamics is still debated since both phosphorylated and non-phosphorylated paxillin have been proposed to regulate lamellipodial protrusions [32, 33], as well as formation [34, 35] and turnover of adhesion formation [21].

Our study identified the striking impact of Rab7 (implicated in transport from early to late endosomes of late endocytic structures/lysosomes) in the regulation of paxillin tyrosine-118 phosphorylation turnover via the autophagy pathway, an evolutionary conserved catabolic process which delivers cytoplasmic cargo to lysosomes via double membrane vesicles called autophagosomes [36] but not through proteasomal degradation [37]. Knockdown of Rab7 but not Rab5 (a regulator of early endosome biogenesis) or Rab11 (involved in perinuclear recycling of endocytosed proteins) (data not shown) prevented 118Y-p-paxillin recruitment and accumulation in the autophagosomes (Figure 4B). As well, we confirmed that the Rab7 mutant lacking GTPase activity failed to rescue118Y-p-Pax recruitment to autophagosomes, highlighting the importance of Rab 7 GTPase activity (Figure 2D), which requires a switch between the GDP-bound off-state and a GTP-bound on-state, necessary for paxillin recognition and recuitment to autophagosomes.

The critical function of Rab7 to recruit autophagy effector FA proteins is of fundamental importance given that autophagy, an evolutionary conserved catabolic process which delivers cytoplasmic cargo to lysosomes via autophagosomes [36], is implicated in the regulation of cell migration [38, 39]. In our study, downregulation of autophagy using both genetic and pharmacological approaches resulted in selective accumulation of 118Y-p-paxillin but not Y31-p-paxillin in autophagosomes (Figure 5A). These results were corroborated by imaging studies confirming intracellular colocalization of 118Y-p-paxillin but not Y31 with LC3 at cell protrusions during FA disassembly (Figure 5C, 5D). In contrast to a previous study showing that mutation of both Y31 and Y118 to non-phosphorylatable amino acids impairs the disassembly of adhesions at FA sites of the leading edge of migrating cells [21, 40], our study revealed a distinct function of Y118, which unlike Y31, can selectively target paxillin to autophagosomes for degradation, triggering FA disassembly.

Paxillin has a proline-rich motif that binds to the Src SH3 domain and Src-FAK kinases, which mediate tyrosine phosphorylation of paxillin [41]. In turn, paxillin interacts with the FAT domain of FAK believed to direct FAK to FA sites. Furthermore, it has been shown that it is the phosphorylated form of paxillin that predominantly recruits FAK into the adhesion sites and that paxillin-induced adhesion turnover occurs in an FAK-dependent manner [40]. To rule out the possibility that paxillin recruitment to autophagosomes is a result of a complex involving Src or FAK, we demonstrated that Src or FAK knockdown had no impact on Y118-p-paxillin recruitment to autophagosomes or interaction with LC3B (Figure 6E, 6F), suggesting that recruitment to autophagosomes occurs in a Src- and FAK-independent manner. In fact, as shown in Supplementary Figure 5, neither FAK, 397Y-p-FAK nor 861Y-p-FAK accumulated in puncta in Rab7-deficient cells, compared to matched control cells.

During selective autophagy, recognition of protein targets involves direct interaction between the substrate and LC3 through LIR motifs, which include cargo receptor proteins such as c-Cbl, NBR1 and p62 [42]. Interestingly, while writing this manuscript a recent study reported that autophagy regulates FA turnover via the cargo protein NBR1 [43]. In our study, using siRNA targeting NBR1 prior to pull down revealed that although NBR1 regulates EGFP-LC3/FLAG-paxillin interaction and colocalizes with 118Y-p-paxillin at FA site, the efficiency of Y118-phospho-paxillin localization to autophagosomes is weaker compared to when manipulating c-Cbl (Supplementary Figure 6). Therefore, we conclude that c-Cbl is a major cargo receptor but not the ubiquitin-binding scaffolding protein p62, which also interacts with LC3 during autophagy. c-Cbl has ubiquitin ligase activity and one study reported that inhibition of c-Cbl ubiquitin ligase activity prevented paxillin interaction and degradation in myocytes [44]. However, in our study we demonstrate that c-Cbl ligase activity had no impact on paxillin/c-Cbl interaction neither on 118Y-p-paxillin expression and turnover (Figure 7D, 7F). In support of our findings, autophagic targeting of active Src is reported to be mediated by c-Cbl independently of c-Cbl E3 ligase activity [23]. Nevertheless, we cannot rule out a contribution of additional posttranslational modifications of paxillin such as serine phosphorylation [37, 45] and ubiquitination [46], both deserving further investigations. Moreover, in agreement with previously published studies, paxillin phosphorylation at Y118 may result in a conformational change in paxillin creating a Crk binding site [30], which in turn possesses a cbl binding domain [47], thereby aiding in paxillin recruitment and detachment from FA complexes. Consistent with recent observations described by Sharifi et al. [15], this is in sharp contrast to what we observed in mouse derived cell lines, where phosphorylation at Y118-p-paxillin was not required for autophagosomal regulation of paxillin and/or its interaction with LC3 in the mouse mammary tumor derived cell line 4T1, or in FAK −/− or SYF −/− mouse embryonic fibroblasts (data not shown), further supporting a tissue-type specific mechanism for this turnover.

In summary, our data provides new mechanistic insights into the role of Rab7 in targeting Y118 site-specific phosphorylation of paxillin to the autophagy pathway to promote focal adhesion turnover (summarized in Supplementary Figure 7) in human breast cancer cells, a process essential for cell locomotion. Noticeable, many of the protein networks involved in these pathways have been reported to be upregulated in many human cancers and can predict invasiveness, these include Rab7 [12] in agreement with another study in melanoma [13], paxillin [48] and Atg proteins [49, 50]. Therefore, molecular mechanisms described herein further enlighten the molecular basis involved in these associations.

## MATERIALS AND METHODS

### Materials

Antibodies used were as follows: anti-118Y-p-paxillin, anti-31Y-p-paxillin and anti-397Y-p-FAK (Invitrogen); anti-GFP, anti-β-actin, anti-Rab7, anti-p62 and anti-LC3B (Santa Cruz); anti-416Y-p-Src and anti-NBR1 (Cell signaling), anti-GFP (Roche); anti-LAMP-1 and anti-HA (Abcam); anti-LC3B, anti-Atg12 and anti-c-Cbl (Novus); anti-Rab5a (BD transduction laboratory); anti-paxillin, anti-Src and anti-FAK (Millipore); anti-LAMP1 (R&D systems); anti-FLAG (Sigma). Anti-mouse and anti-rabbit IgG-peroxidase-conjugated secondary antibodies for Western blot assays were from Bio-Rad. Alexa Fluor594 and 488 conjugated secondary antibodies were from Life Technology. Alexa Fluor 647 conjugated secondary antibodies were from Millipore. Epidermal growth factor was from Gibco; Histodenz (Nycodenz), nocodazole and chloroquine (CQ) were from Sigma-Aldrich. When indicated CQ was used at a concentration of 20 μM, a non-toxic concentration found to induce optimal autophagy inhibition (based on changes in LC3I/II ratio), which is in accordance with previous studies (Sandilands et. al.).

### Cell lines and cell culture

The breast cancer cell lines MDA-231, MCF-7, BT-20 and 4T1 were obtained from the American Type Culture Collection. MDA231-M2 is a highly metastatic variant of MDA-MB-231 cells we isolated from lung nodules induced by the parental MDA-MB-231 [51]. All cell lines were maintained in RPMI 1640 (Fisher) supplemented with 10% FBS and with 1% penicillin and streptomycin antibiotics.

### Plasmids construction

EGFP-paxillin (plasmid #15233), FLAG-paxillin (plasmid #15212), GFP-LC3 (plasmid #22405), mCherry-LC3 (plasmid #40827), GFP-Rab7 (plasmid #12605), GFP-Rab7-T22N (plasmid #12662) were obtained from Addgene. FLAG-paxillin-Y118F was further modified by GeneScript Inc. Primers used for mutation of the EGFP-paxillin and HA-Cbl (kindly provided by Dr. Youcef Yarden, Department of Biological Regulation, The Weizmann Institute of Science, Rehovot, Israel) using site-directed mutagenesis kit (Stratagene): EGFP-paxillin-Y31F: sense, 5′-CAGAGGAAACGCCTTT CTCCTACCAACTGG-3′; antisense, 5′-CCAGTT GGGTA GGAGAAAGGCGTTTCCTCTG-3′, EGFP-paxillin-Y118F: sense, 5′- GAGGAGGAA CACCTGTTCAGCTT CCCAAACAG-3′; antisense, 5′-CTTGTTTGGGAAGCT GAACACGTGTTCCTCCTC-3′ and HA-Cbl-C381A: sense, 5′-GATGGGCTCCACATTCCAACTAGCTAA AA TATGTGCTGAAAATGATA -3′; antisense, 5′-TATCA TTTTCAGCACATATTTTAGCTAGT TGGAATGTGG AGCCCATC-3′. All plasmids were transfected into BT-20 cells using Lipofectamine LTX and PLUS reagents (Invitrogen) according to the manufacturer′s instructions.

### siRNA gene knockdown

Non-targeted siRNA sequence: sense, 5′-UUCUCC GAACGUGUCA CGUdTdT-3′; antisense, 5′-ACGGUG ACACGUUCGGAGAAdTdT-3′, c-Cbl siRNA sequences: sense, 5′-GGGAAGGCUUCUAUUUGUUdTdT-3′; antisense, 5′-AACAAAUAG AAGCCUUCCCdTdT-3′, p62 siRNA sequence: sense, 5′-GCAUUGAAGUUGAUAUCGAUd TdT-3′; antisense, 5′-AUCGAUAUCAACUUCAAUGC dTdT-3′, NBR1 siRNA sequence: sense, 5′-GGAGUGG AUUUACCAGUUAUUdTdT-3′;antisense, 5′-AAUAA CUGGUAAAUCCACUCCdand FAK siRNA sequence: sense, 5′-GCGAUUAUAUGUUAGAGAUAG dTdT-3′; antisense, 5′-CUAUCUCUAACAUAUAAUCGCdTdT-3′ were from Dharmacon. Src siRNA was from Sigma with the following sequences: sense, 5′-CUGAGAGGG CGGUGGGUA UdTdT-3′ (WD04736566); anti-sense, 5′-AUACCCACCGCCCUCUCAGdTdT-3′ (WD04736567). siRNA for Rab5a: sequence 1 (siGENOME siRNA D-004009-01); and sequence 2 (siGENOME siRNA D-004009-02); Atg12: sequence 1 (siGENOME siRNA D010212-03); and sequence 2 (siGENOME siRNA D010212-05) were from Thermo Fisher Scientific. Control siRNA (Cell Signalling) or target siRNA oligonucleotides were expressed in cells by incubated with INTERFERin (Polyplus transfection) in serum-free RPMI medium according to the manufacturer's instruction.

### Generation of stable Rab7-knockdown cells

Stable Rab7 knockdown cells were generated from a polyclonal population as described previously (Benlimame et al., 2005). The target shRNA sequence for Rab7 (GenBank/EMBL/DDBJ, accession no. NM_004637.5) was 5′-CTGCTGCGTTCTGGTATTTGA-3′ (targeting nucleotide 478-498). This sequence was cloned as an inverted repeat into pSUPER.puro vector according to the manufacturer's instructions (OligoEngine).

### Immunoblotting assay

Sub-confluent cells were washed with PBS, lysed in RIPA buffer (50 mM Tris-HCl at pH7.5, 150 mM sodium chloride, 1% tritonX-100, 0.1% SDS, 2mM EDTA and 25mM sodium fluoride) supplemented with 1mM PMSF and protease inhibitor cocktail (Roche) for 10 min on ice and centrifuged (13,000 rpm at 4°C for 20 min) to separate cell lysate. Cell lysate (50 μg protein, as measured by the Bradford protein assay) was then added with SDS sample buffer (Tris at pH 6.8, 20% glycerol, 5% SDS, bromophenol blue and β-mercaptoethanol) and boiled for 5 min. Samples were then resolved through 13% SDS-PAGE gels, transferred to nitrocellulose membrane, blotted with primary antibodies at different dilution in cold room overnight, and then amplified with horseradish peroxidase-conjugated secondary antibodies for 1h in room temperature and enhanced by chemiluminescence detection system.

### Immunoprecipitation assays

Cells transfected with both FLAG-paxillin (or FLAG-paxillin-Y118F) and GFP-LC3 were washed with PBS and lysed in lysis buffer (50 mM Tris-HCl at pH 8.0, 50 mM sodium chloride, 0.5% tritonX-100, 0.5% 2 NP-40, 0.5 % deoxycholate, 0.1% SDS and 5 mM EDTA) supplemented with 1mM PMSF and protease inhibitor cocktail (Roche) for 10 min on ice and cell lysate isolated following centrifugation (13,000 rpm at 4°C for 20 min). Cell lysate (1000 μg protein, measured by bradford protein assay) was immunoprecipitated with either 2 μg anti-FLAG or anti-GFP antibodies or IgG (sigma) overnight at 4°C, and then incubated with protein G magnetic beads (Millipore) for 1 h at 4°C. Beads were washed three times with IP buffer (50 mM Tris-HCl at pH 8.0, 50 mM sodium chloride, 1% tritonX-100 and 5 mM EDTA), then added with SDS sample buffer (Tris at pH 6.8, 20% glycerol, 5% SDS, bromophenol blue and β-mercaptoethanol) and boiled for 5 min, then separated by 10% SDS-PAGE. For interaction between c-Cbl and paxillin, cells were transfected with HA-Cbl (or HA-Cbl-C381A) and FLAG-paxillin and pulled down using anti-FLAG antibody.

### Density gradient centrifugation for autophagosome enrichment and characterization

Cells (1 × 10^8^) were washed with PBS and resuspended in 1 ml cold buffer A (250 mM, 10mM HEPES, pH 7.4, 1 mM EDTA) supplemented with 1mM PMSF and protease inhibitor cocktail (Roche). Cells were disrupted by repeated aspiration through 26 gauge needle and centrifuged (2,000 rpm at 4°C for 10 min) and 0.95 ml supernatant was diluted with 1.45 ml 85.6% Nycodenz (Sigma) solution to become 52%, then placed on the bottom of the 13 ml ultra-clear centrifuge tube (Beckman). A discontinuous Nycodenz gradient (26%, 24 %, 20%, 15%) was layered on the top of the 52% supernatant and centrifuged at 24,700 rpm at 4°C for 3 h in a SW41 rotor (Beckman). Fractions were washed with cold PBS and collected as indicated (A1, A2, L, M). Pellets were collected at 14,800 rpm, 4°C for 1 h and used for immunoblotting experiments.

### Immunofluorescence microscopy

Cells were grown on 18-mm cover glass coated with 5 μg/ml fibronectin for 24 h in 4°C, placed in 6-well culture plate for 24 h, rinsed in PBS, fixed with 4% paraformaldehyde/PBS for 10 min, washed twice in PBS with 0.2% TritonX-100, blocked in PBS with 0.2% Triton X-100 containing 1% BSA (Bioshop) and incubated with primary antibodies overnight in 4°C (all in 1/100 diluted by blocking solution). The cells were washed with PBS containing 0.2% TritonX-100 and subsequently incubated with Alexa Fluor 488-labelled, Alexa Fluor 594-labelled, or Alexa Fluor 647-labelled secondary antibodies (all were used at 1/500 dilution in blocking solution) for 1 hr in room temperature. The nuclei were stained with DAPI (0.1 μg/ml) for 5 min before mounted with aqueous mounting medium. Cells were imaged using WaveFX spinning disk confocal microscope system (Quorum Technologies INC.). Images shown are representative of three independent experiments

### Live cell imaging microscopy

For the monitoring of FA turnover, cells were transfected with EGFP-paxillin and plated in RPMI 1640 serum-free medium on a multiwell chambered coverglass (LabTek) prepared by coating overnight in 4°C with 5 μg/ml fibronectin. The coverglass was directly placed on a heated stage with 5% CO_2_ and stimulated with 20 ng/ml epidermal growth factor. Fluorescent images were captured every 5 min for 2 h using a heated 64 X NA 1.40 objective at WaveFX spinning disk confocal microscope system (Quorum Technologies INC.). Fluorescence intensities of individual adhesions were measured over time by Volocity imaging software (PerkinElmer) and quantified according to the previously described protocol (Franco et. al 2004b; Webb et al., 2004). Measurements were made on at least 20 individual adhesions in four separated cells for both Rab7 silenced and control groups. For investigating colozalization, cells were transfected with both GFP-paxillin and mcherry-LC3, then plated in RPMI 1640 medium with 10% FBS on a multiwell chambered coverglass (LabTek). Fluorescent images were captured every 15 sec for 2 h using a heated 63 X NA 1.40 objective using a WaveFX spinning disk confocal microscope system.

### Nocodazole-based FA disassembly assay

FA disassembly and reformation, cells were grown on glass-coverslips coated with 5 μg/ml fibronectin for 24 h in 4°C, serum starved for 4 h in RPMI1640 serum free medium and then treated with 2μM nocodazole in serum-free medium for 2 h to allow a complete depolymerization of microtubules. Nocodazole was washed-out with serum-free medium and cells were further incubated at 37°C to allow a reformation of FA structure. Subsequently, cells were fixed and processed for immunofluorescence staining at different time intervals. Cells were stained with Rab7, 118Y-p-paxillin and LC3B. Focal adhesion dynamics was analyzed as previously described [22] using Image J software.

### Live cell locomotion assay

Cells were seeded at very low density in serum-free medium on a multiwell chambered coverglass (LabTek) prepared by coating overnight in 4°C with 5 μg/ml fibronectin. The coverglass was placed on a heated stage with 5% CO_2_ and stimulated with 20 ng/ml epidermal growth factor before monitoring the cell movement using a heated 40 X NA 1.40 objective at WaveFX spinning disk confocal microscope system (Quorum Technologies INC.). Single cell speed was determined by manually tracing the cell periphery every 20 min for 2 h by using Volocity imaging software (PerkinElmer).

### *In vitro* statistical analysis

All experiments counting 118Y-p-paxillin and LC3 puncta were performed in triplicate with similar results. Quantitative data in single cell migration experiments and puncta quantification were performed in 15 separate cells for both Rab7 silenced and control cells. For FA disassembly and FA life time quantification, measurements were made on at least 40 focal adhesions in 10 separate Rab7 silenced and control cells. All data are presented as mean ± SEM using Prism (GraphPad Software). Statistical significance was analyzed using unpaired two-tailed Student's *t* test. Data were deemed to be statistically significant if *P* < 0.05. Error bars indicate SEM unless otherwise indicated.

### *In vivo* xenograft model of breast cancer metastasis

All experiments were carried out according to protocol number 4101 of the McGill University Animal Care Committee. MDA231-M2 cells (1 × 10^6^ cells/ each mouse) expressing scramble shRNA and their matched cells stably expressing Rab7 shRNA were transplanted into the mammary fat pad of SCID mice. Tumor size was measured using a caliper, and tumor volume was calculated as π/6 (length × width^2^). Primary tumors were excised once they had reached a mean size of 0.8 cm^3^. The wound was closed with a single layer of surgical clips. Mice were sacrificed 40 days after transplanting. The lungs were fixed in 10% Bouin's fixative and lung metastatic nodules on the surface were counted using a stereomicroscope (Optimax; Leica). Statistical comparison between groups was performed using a computer-based statistical package from Statistical Product and Service Solution (Chicago, IL) as we described earlier [52].

## SUPPLEMENTARY MATERIALS FIGURES AND VIDEOS






