# Supplementary material for: Endosomal sorting and c-Cbl targeting of paxillin to autophagosomes regulate cell-matrix adhesion turnover in human breast cancer cells
Source: Oncotarget. 2017 Mar 10;8(19):31199–214. doi: 10.18632/oncotarget.16105 (PMC5458201; doi:10.18632/oncotarget.16105)
Supplement: Supplementary file 1 [file oncotarget-08-31199-s001.pdf]

Endosomal sorting and c-Cbl targeting of paxillin to autophagosomes regulate cell-matrix adhesion turnover in human breast cancer cells

Supplementary Materials

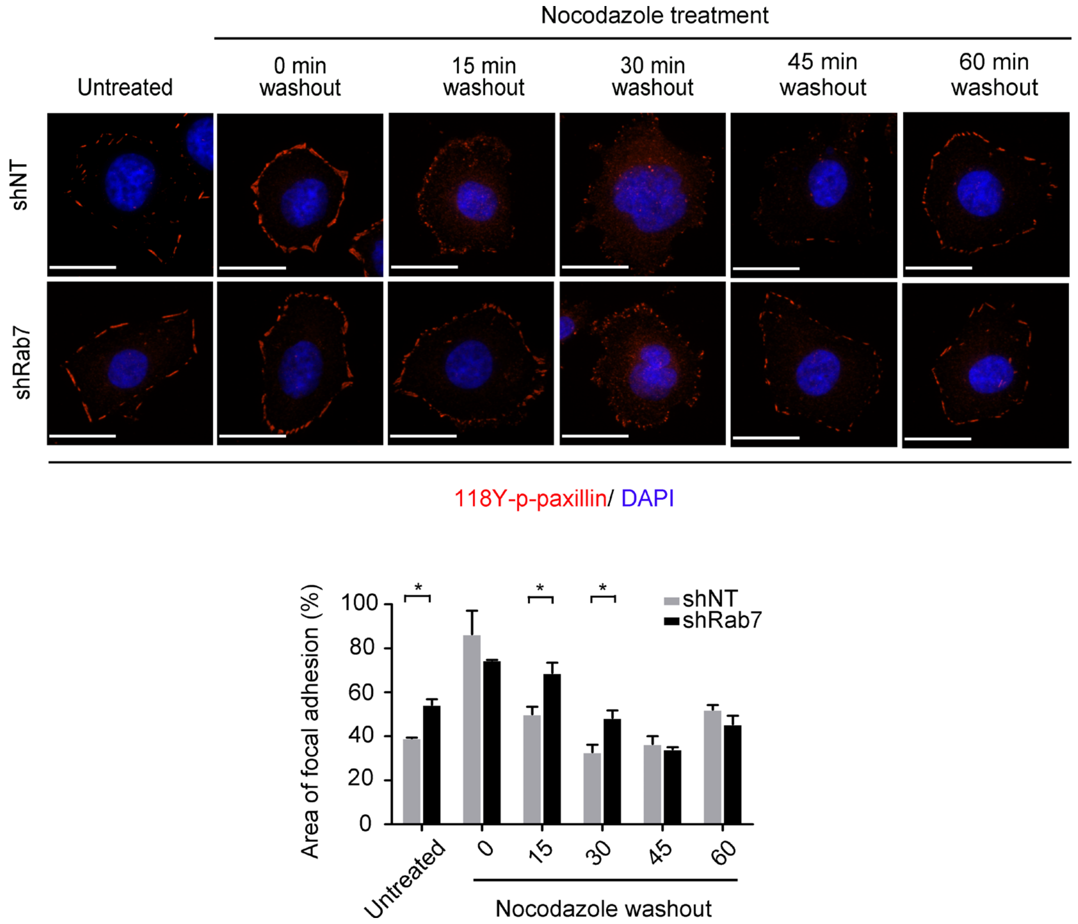

Supplementary Figure 1: Knockdown of Rab7 attenuates focal adhesion turnover. Top, serum-starved control (shNT) and Rab7-silenced (shRab7) BT-20 cells were incubated with 2 µM nocodazole (NZ) for 2 h, fixed at indicated time points after washing out NZ and immunobloted with anti-118Y-p-Pax (red) and DAPI (blue). Scale bar, 20 µm. Bottom, FA area of control (shNT) and Rab7-silenced cell (shRab7) were quantified by Image J software. Data are presented as mean ± SEM (**P* < 0.05, *n* = 3).

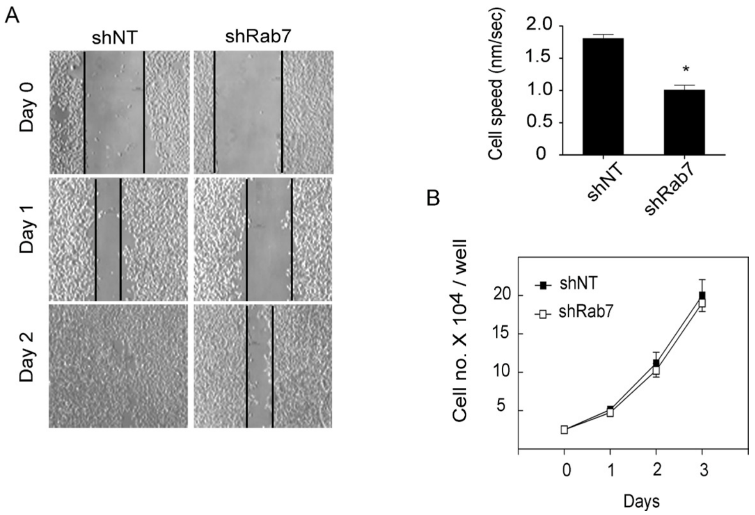

Supplementary Figure 2: Inhibition of autophagy stabilizes BT-20 cells motility but has no impact on cell survival. (**A**) Left, cell migration of BT-20 cells on control (shNT) and Rab7-silenced (shRab7) groups were analyzed by wound-healing assay. BT-20 cells were scratched and monitored over 48 h to determine the wound closure. Scale bar, 2 mm. Right, the speed of BT-20 cells shNT and shRab7 were quantified. Data are presented as mean ± SEM (**P* < 0.05) (*n* = 3). (**B**) Control (pSR) and Rab7-silenced (shRab7) BT-20 cells were plated and then counted the number of cells at different days. Graph shows the mean of total number of cells ± SEM (**P* < 0.05, *n* =3).

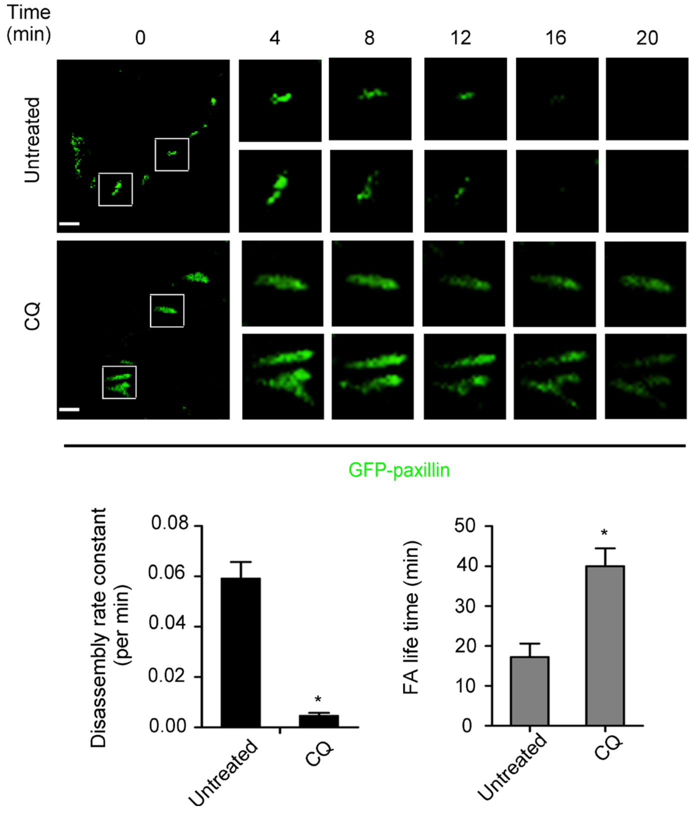

Supplementary Figure 3: Inhibition of autophagy maturation by chloroquine attenuates focal adhesion turnover. Top: Serum-starved BT-20 cells were transfected with GFP-paxillin for 24 h, treated with PBS (untreated) or 20 μM chloroquine (CQ) for 24 h, stimulated by 20 ng/ml EGF and focal adhesion (FA) turnover was analyzed by time-lapse spinning disc microscopy. Scale bar, 2 μm. Higher-magnification images of the inserts are shown indicating positions of paxillin-containing FAs. Bottom: quantification of FA disassembly rate and FA life time of untreated and CQ-treated cells (mean ± SEM, *n* = 10, **p* < 0.05).

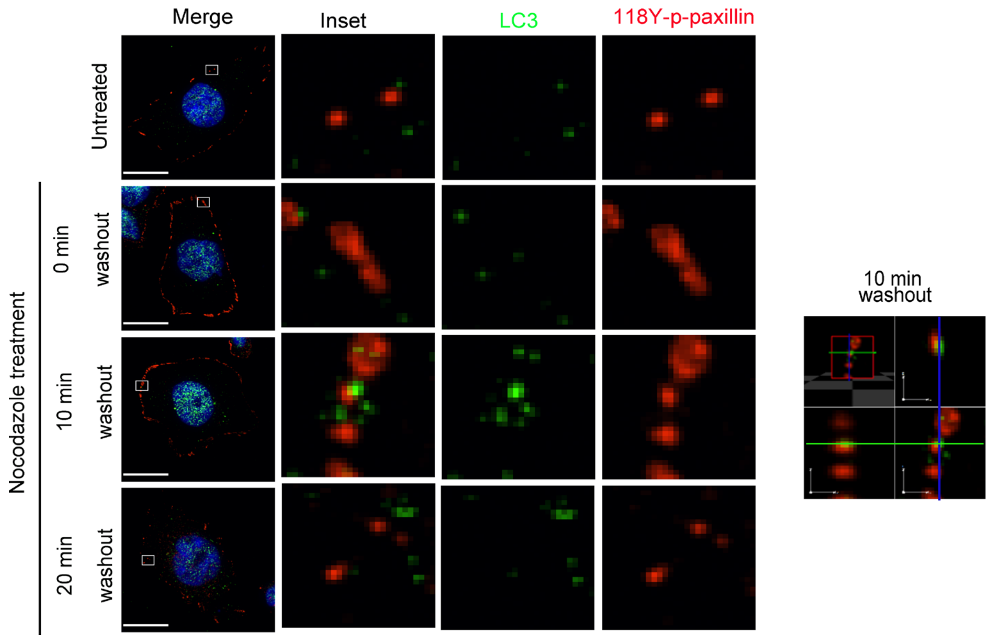

Supplementary Figure 4: LC3 transiently interacts with Y118 phosphorylated paxillin at FA site. Serum-starved BT-20 cells were incubated with either DMSO or 2 µM nocodazole (NZ) for 2 h, fixed at 0 min, 10 min or 20 min after washing out NZ and immunostained with anti-118Y-p-paxillin (red), anti-LC3 (green) antibodies and DAPI (blue). Scale bar, 20 µm. Higher magnification images of the inserts indicate colocalization of LC3 and 118Y-p-paxillin at FA regions.

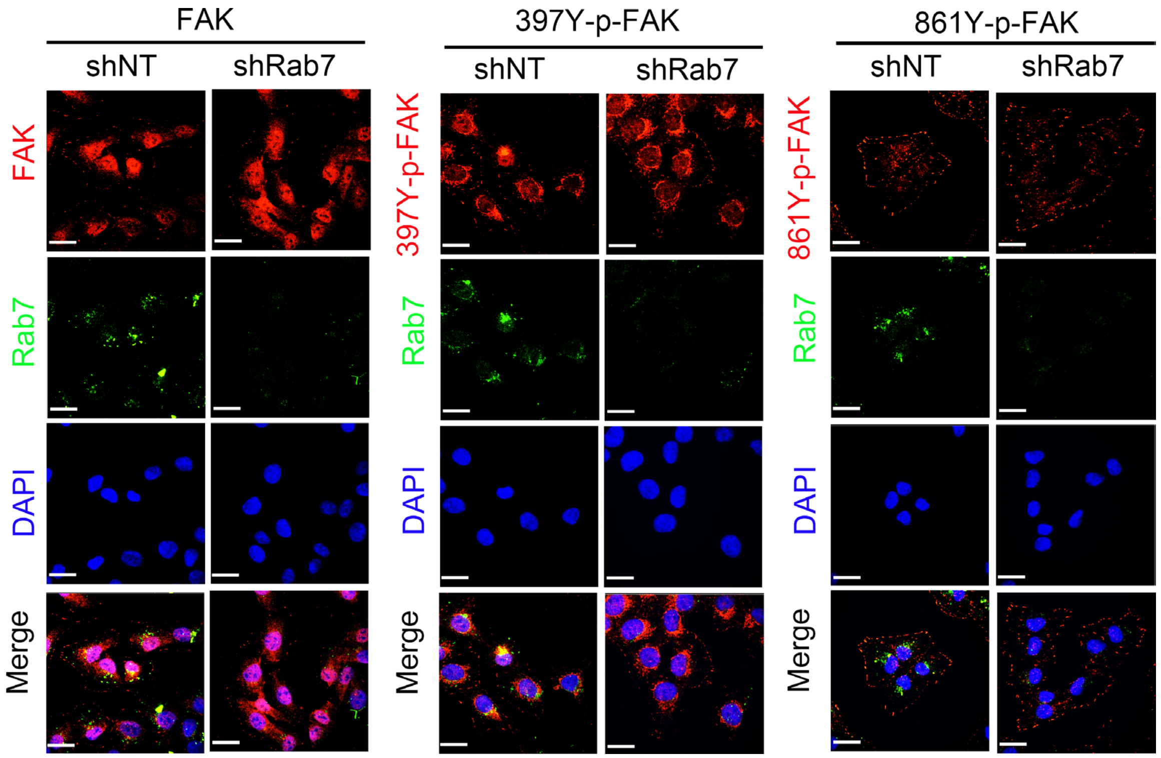

Supplementary Figure 5: FAK and its phosphorylated form do not accumulate in autophagosomes following Rab7 knockdown. BT-20 cells expressing shNT or shRab7 were fixed and stained for anti-FAK, anti-397Y-p-FAK antibody or 861Y-p-FAK (red), anti-Rab7 antibody (green) and with DAPI (blue). Scale bar, 20 µm

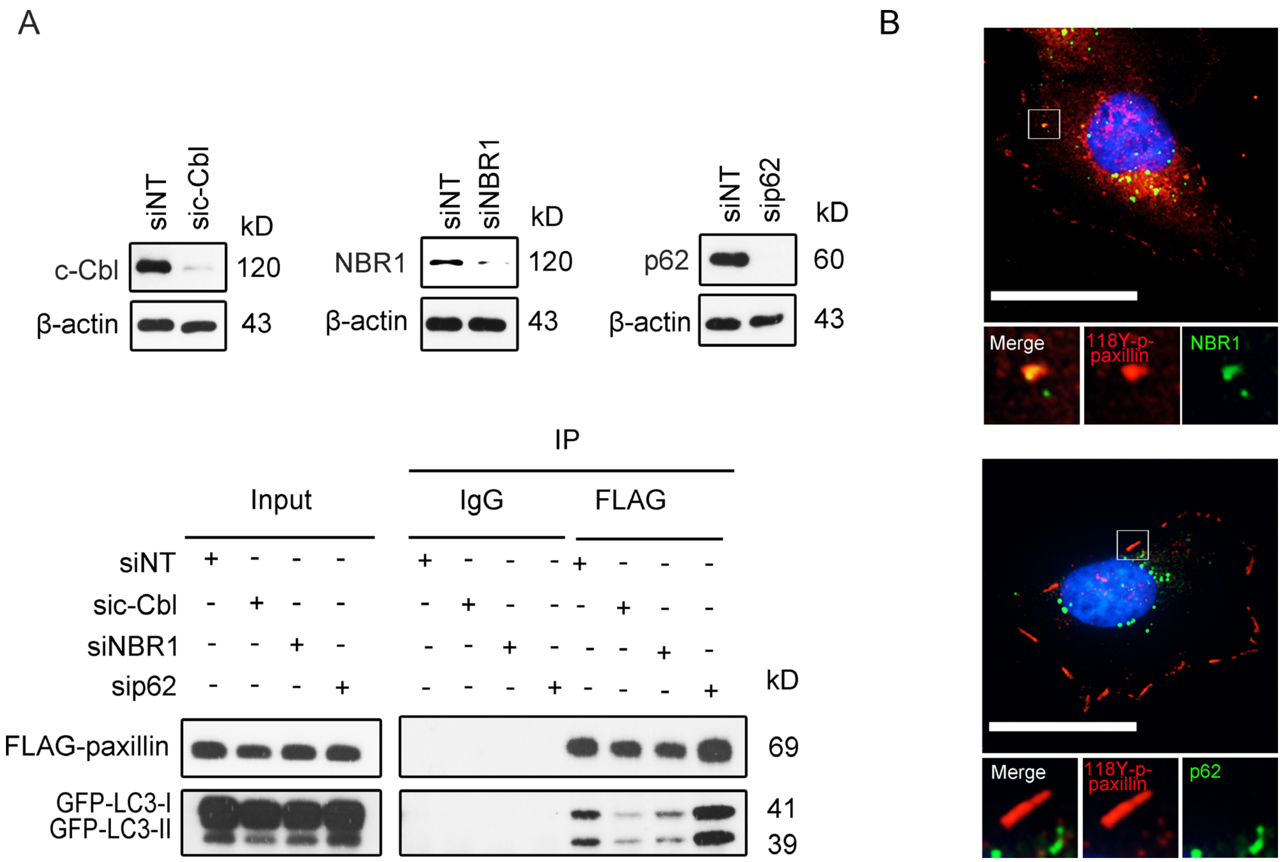

Supplementary Figure 6: c-Cbl and NBR1 but not p62 modulates paxillin/ LC3 complex formation. (**A**) Left: BT-20 cells were transfected with either non-targeted (NT), c-Cbl, NBR1, p62 siRNA for 48 h, then immunoblotted with anti-c-Cbl, anti-NBR1, anti-p62 and anti-β-actin antibodies. Bottom: BT-20 cells were transfected with NT, c-Cbl, NBR1 or p62 siRNA for 24 h and then transfected with FLAG-paxillin and GFP-LC3 plasmids for another 24 h and immunoprecipitated by either anti-IgG or anti-FLAG antibodies. Lysates were then immunoblotted with anti-FLAG and anti-GFP antibodies. (**B**) BT-20 cells were fixed and stained for anti-118Y-p-paxillin antibody (red), anti-NBR1 or p62 antibody (green) and with DAPI (blue). Scale bar, 20 µm. Enlargements of the boxed regions are also shown indicating association of 118Y-p-paxillin and NBR1 or p62.

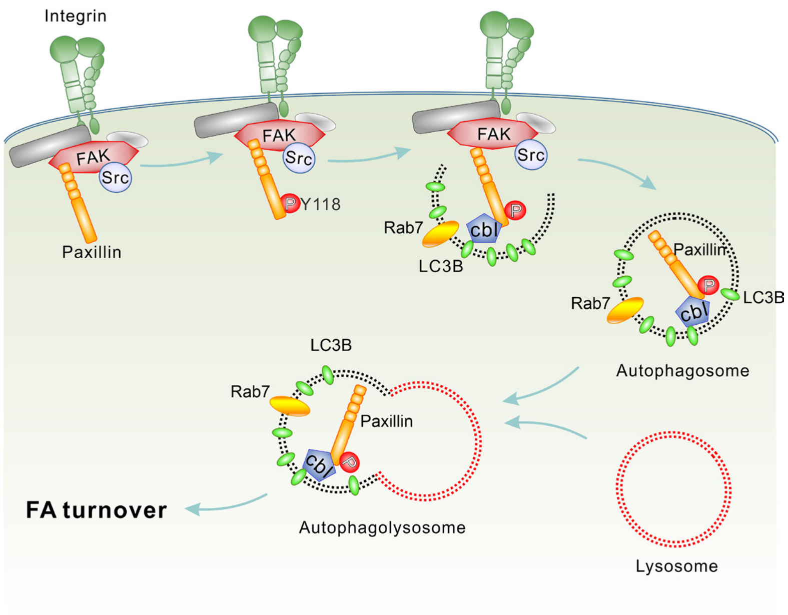

Supplementary Figure 7: A schematic summary depicting the Rab7-mediated focal turnover regulated by Y118-phosphorylated paxillin via the autophagy pathway. Paxillin is a scaffold protein involved in the formation of FA complexes and activated by FAK/Src kinases following integrin activation, in part via interaction with FAK FAT domain. Phosphorylation of paxillin on Y 118 creates a binding site for c-Cbl E3 ubiquitin ligase through its LIR motif, which subsequently promotes paxillin interaction with the mammalian Atg8 homolog LC3 to direct Y118 paxillin processing through autophagy resulting in FA turnover. During this process, Rab7 serves as an important regulator to mediate late autophagosome to fusion with lysosome.

Supplementary Video 1: Single-cell tracking of migrating shNT and shRab7 cells. Spinning disk confocal microscopy of single cell migration of shNT (left) and shRab7 (right) expressing BT-20 cells. Images were acquired every 7 min. The video plays at 5 frames per second and is accelerated approximately 360 fold. This video is related to Figure 1C. See Supplementary_Table_1

Supplementary Video 2: Dynamics of mCherry-LC3 to EGFP-paxillin-WT and EGFP-paxillin-Y118F at cell protrusion. Spinning disk confocal microscopy of mCherry-LC3 (red) targeted to EGFP-paxillin-WT (green) (left) and non-targeted to EGFP-paxillin-Y118F (green) (right) at cell protrusion. Imaged were acquired every 15 sec. The video plays at 5 frames per second and is accelerated approximately 36 fold. This video is related to Figure 5C. See Supplementary_Table_2
